# Execution of a participatory supportive return to work program within the Dutch social security sector: a qualitative evaluation of stakeholders’ perceptions

**DOI:** 10.1186/s12889-016-2997-x

**Published:** 2016-04-14

**Authors:** Lieke Lammerts, Frederieke G. Schaafsma, Willem van Mechelen, Johannes R. Anema

**Affiliations:** Department of Public and Occupational Health, EMGO Institute for Health and Care Research, VU University Medical Center, P.O. Box 7057, Amsterdam, MB 1007 Netherlands; Research Center for Insurance Medicine, AMC-UMCG-UWV-VUmc, Amsterdam, Netherlands

**Keywords:** Return to work, Complex intervention, Stakeholders’ perceptions, Qualitative study, Context, Setting, Social security, Common mental disorders, Temporary workers, Unemployed workers

## Abstract

**Background:**

A process evaluation of a participatory supportive return to work program, aimed at workers without a (permanent) employment contract who are sick-listed due to a common mental disorder, revealed that this program was executed less successfully than similar programs evaluated in earlier studies. The program consisted of a participatory approach, integrated care and direct placement in competitive employment. Aim of this study was to get a better understanding of the execution of the program by evaluating stakeholders’ perceptions. In the absence of an employer, the program was applied by the Dutch Social Security Agency, in collaboration with vocational rehabilitation agencies. Together with the sick-listed workers, these were the main stakeholders. Our research questions involved stakeholders’ perceptions of the function(s) of the program, and their perceptions of barriers and facilitators for a successful execution of the program within the Dutch social security sector.

**Methods:**

Semi-structured interviews were held with five sick-listed workers, eight professionals of the Social Security Agency, and two case managers of vocational rehabilitation agencies. Interview topics were related to experiences with different components of the program. Selection of respondents was based on purposive sampling and continued until data saturation was reached. Content analysis was applied to identify patterns in the data. Two researchers developed a coding system, based on predefined topics and themes emerging from the data.

**Results:**

Although perceived functions of some components of the program were as intended, all stakeholders stressed that the program often had not resulted in return to work. Perceived barriers for a successful execution were related to a poor collaboration between the Dutch Social Security Agency, vocational rehabilitation agencies and healthcare providers, the type of experienced (health) problems, time constraints, and limited job opportunities.

**Conclusions:**

For future implementation of the program, it will be important to consider how a better integration of services by the Dutch Social Security Agency, vocational rehabilitation agencies and the mental healthcare sector can be improved in order to address treatment and vocational needs simultaneously, and to better match the sick-listed worker with the limited opportunities in the Dutch labor market.

**Trial registration:**

NTR3563

**Electronic supplementary material:**

The online version of this article (doi:10.1186/s12889-016-2997-x) contains supplementary material, which is available to authorized users.

## Background

Complex interventions consist of multiple interacting components [1, 2]. When studying its effectiveness in a randomised controlled trial (RCT), it is often difficult to determine which components have caused an effect. Insight into the execution of these components in the study’s practice helps to interpret the results of a RCT [2, 3], and to improve the feasibility of the intervention for future implementation [2]. Therefore, process evaluations alongside RCTs have become more common [4].

In an earlier study we conducted a process evaluation of a participatory supportive return to work (RTW) program, alongside a RCT, using quantitative research methods [5]. Aim of the participatory supportive RTW program was to improve RTW of workers without a (permanent) employment contract, sick-listed due to a common mental disorder (CMD). These workers often face a greater distance to the labor market compared to sick-listed permanent employees, as many of them have no workplace to return to [6]. The program was evaluated within the Dutch Social Security sector. In the Netherlands, sick-listed workers who have no (longer an) employer are entitled to occupational healthcare (OHC) by the Dutch Social Security Agency (SSA). The core of the program consisted of a participatory approach in which the sick-listed worker was encouraged to identify obstacles for RTW and to think of solutions and suitable work, in cooperation with a RTW coordinator of the Dutch SSA. This was monitored by a labor expert, whose responsibility it was to reach consensus between the sick-listed worker and the RTW coordinator and to summarize the proposed solutions and suggestions for suitable work in a RTW action plan. In order to agree upon RTW possibilities and to avoid conflicting advice to the sick-listed worker, the insurance physician of the SSA applied an integrated care approach by contacting the healthcare provider(s) of the sick-listed worker directly after the medical assessment. Vocational rehabilitation agencies were contracted in order to place the sick-listed worker in a suitable competitive job, based on the RTW action plan.

Findings of our process evaluation revealed that the participatory supportive RTW program was executed less successfully compared to similar programs evaluated in earlier studies [5]. A small part of the intervention participants actually started with the program. In many other cases the insurance physician assessed a contra-indication for participation in the program. In these cases, the program was not considered suitable. Only for half of the sick-listed workers that actually followed the program, application of an integrated care approach was reported, and only two sick-listed workers were placed in a suitable competitive job. Often the program was not executed in accordance with the prescribed time-table. Nevertheless, overall satisfaction with the participatory approach was good [5].

The aim of this present study was to get a better understanding of the execution of the participatory supportive RTW program, by evaluating the execution of the intervention in relation to its setting. Several authors state that to account for the complexity of an intervention, it is not only important to quantify what happened in practice, but also to identify (contextual) factors that could have influenced the execution of an intervention [7–10], and to better understand the function of an intervention within its setting [11]. To illustrate, cultural expectations stemming from the beliefs, attitudes and experiences of stakeholders [7], staffing issues, such as time and resource difficulties or competing priorities, and organizational changes [8] are all factors that could influence the execution of an intervention. Vice versa, the very fact that the intervention is being conducted in a particular setting, could also change that setting. Often it is difficult to disentangle the intervention from its setting [10] and it may even be undesirable to do so. Hawe et al. [11] explain that a complex intervention could look different across different settings, but could still have the same function(s). This means that when evaluating the execution of a complex intervention the question should be whether the intervention and its separate components have had the intended function rather than only how the intervention looked like in practice. In this study we evaluated the function(s) of the participatory supportive RTW program within the Dutch social security sector and we investigated barriers and facilitators for a successful execution of the program.

Qualitative study methods are considered useful for unravelling processes of change, exploring responses to the intervention and describing the intervention as executed in practice [12]. For that reason, we decided to conduct interviews with the main stakeholders of the participatory supportive RTW program, i.e., sick-listed workers, professionals of the Dutch SSA and professionals of contracted vocational rehabilitation agencies. These stakeholder groups all represent different interests in the OHC field. Also their perceptions of RTW interventions are likely to differ, as can be illustrated by the studies of Tiedtke et al. [13] and Maiwald el al. [14]. To be able to reflect on (the influence of) different perspectives of the participatory supportive RTW program, and to get a broad understanding of the execution of the program, members of all main stakeholder groups were involved in our evaluation. Our main research questions were: what were stakeholders’ perceptions of the function(s) of the participatory supportive RTW program? And what were their perceptions of barriers and facilitators for a successful execution of the program within the Dutch social security sector?

## Methods

### Design

The study design consisted of a qualitative study that was conducted alongside a RCT, titled ‘the Co-WORK study’. The aim of the Co-WORK study was to investigate the (cost-)effectiveness of the participatory supportive RTW program in comparison with usual OHC by the Dutch SSA. The Medical Ethics Committee of the VU University Medical Center (Amsterdam, The Netherlands) gave Ethical approval for the study. The same committee declared that no comprehensive ethical review was needed for this qualitative study. The trial was registered at the Dutch Trial Register (‘Nederlands Trial Register’) on August 7, 2012 (NTR3563). All participants signed informed consent. More information about the trial can be found in the study protocol [15].

### Study setting

The participatory supportive RTW program was aimed at unemployed workers, temporary agency workers and fixed-term contract workers who had filed a sickness benefit claim at the Dutch SSA, with mental health problems as main reason for their sickness benefit claim. Other stakeholders in the intervention were insurance physicians, labor experts and RTW coordinators of the Dutch SSA, and case managers of contracted vocational rehabilitation agencies. Seven SSA front offices participated in the program, located in the western, central and eastern region of the Netherlands, and three vocational rehabilitation agencies, operating on a national level.

### Selection and recruitment of respondents

To get a broad understanding of perceived functions of the program and perceived barriers and facilitators for a successful execution, we wanted to identify all different perceptions of stakeholders in our study. We used purposive sampling to select stakeholders with various characteristics, as we expected that their perceptions of the execution of the program could differ. In the remaining of this article sick-listed workers who participated in the participatory supportive RTW program are referred to as ‘clients’, as at least some of them were no longer sick-listed at the timing of the interviews.

Clients were selected on the basis of a variation in gender, educational level, age, duration of last employment, region and date of enrolment in the Co-WORK study. This information was collected during the baseline measurement of the Co-WORK study. Clients were matched to a SSA front office for RTW guidance, based on their zip code. By selecting clients from different regions they automatically belonged to different SSA front offices. We only selected clients that had actually participated in the participatory supportive RTW program. We also selected insurance physicians, labor experts and RTW coordinators from different participating SSA front offices. Moreover, these professionals had to have applied the participatory supportive RTW program at least twice, so that perceptions were not based on only a single case. As at each SSA office a maximum of two professionals of each profession participated in the program, further selection based on other characteristics was not possible. This was also the case for the vocational rehabilitation agencies. Of the three participating agencies, one case manager was selected for an interview.

For the recruitment of respondents we used communication methods that had been used before for contacting the different stakeholders during the Co-WORK study. Clients were invited for an interview by telephone. During this telephone conversation, they were informed about the purpose of the interviews, the content and duration of the interviews, and other study procedures. In case someone was willing to participate, an appointment for an interview was made directly. A confirmation of this appointment was sent to the client by postal mail, including a summary of the study procedures and an informed consent form. By signing informed consent, the client agreed with his participation in the study and with the recording of the interview. Professionals were invited for participation by e-mail. In this e-mail all study procedures were explained. By responding to the e-mail and expressing their willingness to participate, professionals consented to their participation in the study.

Three clients that were approached for participation in an interview declined. Further, one insurance physician and one case manager did not respond to the invitation. Selection and recruitment of respondents for the interviews was continued, until data saturation was reached. Data saturation was considered to be reached when a new interviewee within a stakeholder group described to a large extent the same functions of the participatory supportive RTW program and/or the same barriers and facilitators for a successful execution of the program, compared to earlier interviewees within the same stakeholder group.

### Study population

In total, 15 respondents were included in this study. Interviews were held with two insurance physicians, three labor experts, three RTW coordinators, two case managers of vocational rehabilitation agencies and five clients. Professionals were from four different SSA front offices and two vocational rehabilitation agencies. Clients belonged to four different SSA front offices. More background information on the clients can be found in Table 1.

**Table 1:** background information clients ^a^

	Gender	Age ^a^	Education ^ab^	Duration of last employment (years) ^a^	Time between start in Co-WORK and interview (months)
Client 1	Female	55	High	3	13
Client 2	Male	54	Low	13	24
Client 3	Male	43	Low	0.5	18
Client 4	Female	43	Middle	6	12
Client 5	Female	29	Middle	0.7	17

^a^ Measured at baseline of the Co-WORK study

^b^ Low educational level included no education, primary school or lower vocational education; middle educational level included intermediate vocational education or secondary school; high educational level included higher vocational education or university

### Interviews

The interviews were conducted by telephone by L.L., the first author of this study. The semi-structured interviews took 20–45 min, dependent on the number of topics discussed. Prior to the interviews a topic-list was created for each group of respondents or stakeholders. This topic list contained both general topics and more specific questions about experiences with different components of the participatory supportive RTW program. Examples of general topics were ideas about the program’s effectiveness and points of improvement. The more specific topics were related to the specific role of the respondent in the program, and differed between stakeholders. Insurance physicians were asked about contra-indications for participation in the program and about their experiences with the application of an integrated care approach. Labor experts and RTW coordinators were asked to evaluate the use of a participatory approach. Specific topics for the case managers of the contracted vocational rehabilitation agencies included ways in which was searched for suitable competitive jobs and their effectiveness. Clients were asked to evaluate all different components of the program, i.e., integrated care, a participatory approach and direct placement in a competitive job. Table A1 (*S*ee Additional file 1) gives an overview of the topics that were discussed during the interviews. Each interview was recorded and fully transcribed (verbatim).

### Analysis

Interviews were analyzed according to the main principles of content analysis. This means that the interview transcripts were analyzed through a systematic classification process of coding and identifying themes or patterns in order to describe the execution of the RTW program in practice [16]. Our aim was to identify perceived functions of the program and its separate components, and perceived barriers and facilitators for a successful execution. We wanted to get new insights and to relate this to existing knowledge. To reach these purposes, we used techniques from both directed and conventional content analysis. Initial codes were directed by the topic list, conform the principles of directed content analysis. Sub codes emerged from the data and were used to express meanings or themes, as is common in conventional content analysis [16]. Methods used for conventional content analysis are similar to the grounded theory (GT) approach, although the GT approach goes beyond content analysis to develop theory [16]. The analysis was done in multiple phases, consisting of open coding, axial coding and selective coding, based on the GT approach [17].

Two researchers performed the analysis. First, a list of initial codes was created by L.L.. Then, six transcripts were coded by L.L. and a research assistant, J.O. independently, with the use of ATLAS.ti 7.1.8. During this phase of open coding, the transcripts were carefully read and divided into text parts. Text parts that seemed relevant, were coded by using the initial codes and creating (sub) codes. In this way, both researchers created an extended code list. During the phase of axial coding, the code lists were discussed by both researchers in order to reach consensus about a provisional list of codes and the interpretation of these codes. During this consensus meeting, it was carefully assessed whether the created codes were appropriate to describe the data and whether the text parts were given the most suitable code. The relation between main and sub codes was discussed, codes describing the same themes were clustered, and codes describing multiple themes were split into different codes. After consensus was reached, all transcripts were (again) analyzed by L.L, using the provisional code list. When necessary, new codes were created. Finally, patterns in the data were identified by looking for returning themes and by making connections between these themes. During this phase, we identified perceived functions of the participatory supportive RTW program and of its separate components, and perceived barriers and facilitators for a successful execution if the program. Codes describing the functions of the program were mostly directed by the predefined topic list. Barriers and facilitators mostly emerged from the data. All authors were involved in this phase of selective coding.

We used quotes originating from the interviews to illustrate our findings. Cited professionals were described by the job title of their profession. For clients, we used numbers (1–5), corresponding to the numbers used in Table 1. Numbers were also used to differentiate between two or more respondents with the same profession, when multiple quotes were used to illustrate one particular finding.

## Results

We present stakeholders’ perceptions of (1) functions of the participatory supportive RTW program, (2) barriers for a successful execution of the program, and (3) facilitators for a successful execution of the program. We distinguished between perceived functions of the program’s separate components, i.e., integrated care, a participatory approach and direct placement in a competitive job. Subsequently, frequently mentioned barriers and facilitators for a successful execution of the program were summarized. We distinguished between perceptions by different stakeholders, when they had different points of view.

### Perceived functions of the participatory supportive RTW program

#### Perceived functions of integrated care

The insurance physicians thought that the communication and cooperation between them and the clients’ healthcare provider(s) had improved after they had contacted the healthcare provider(s).

*Insurance physician 1: “I believe that working together for a client can have a positive effect, because it leads to respect for each other’s discipline.”*

*Insurance physician 2: “It leads to interaction, while normally you ask for information and that’s it.”*

Another perceived function of this component of the participatory supportive RTW program was a shift in paradigm by the healthcare providers from a disability-oriented approach to an approach in which work became more central.

*Insurance physician: “My experiences were positive, because the healthcare providers became aware of the clients’ participation in the program and also responded positive to the focus on work resumption. So, this had opened the healthcare providers’ eyes and they were no longer solely focused on the health complaints of their clients.”*

However, the insurance physicians not always thought it was necessary to contact the clients’ healthcare provider(s). One of the insurance physicians stressed that only sick-listed workers with mild (mental health) problems participated in the participatory supportive RTW program. Therefore, no conflicting advice could be expected. In case of more severe problems, the insurance physician would not have the client start with the program.

The clients indicated that work resumption was barely discussed with their healthcare providers. They also had not received any conflicting advice from their healthcare providers regarding their possibilities for RTW.

*Client 4: “Of course he understood my situation. He told me: ‘You’re not fit. You’re not at your best. You should realize that your chances of getting hired are extremely small.”*

#### Perceived functions of a participatory approach

The RTW coordinators and labor experts thought that it was important to actively involve the client in the creation of a RTW action plan and also believed that this participatory approach had actually led to a more active participation in vocational rehabilitation by the client.

*Labor expert:* “*Normally we ask clients about their background and we discuss some obstacles, but then we mainly speak about limitations that were noticed by the physician. Now, clients had to come up with their own ideas about obstacles and suggestions to overcome these obstacles. This self-reflection was hard, but it helped to get them in another mindset.”*

According to the labor experts and RTW coordinators, many clients were strongly involved in the identification of obstacles and finding solutions and suitable work. However, they also thought that the input of the clients varied.

Another function of the participatory approach, perceived by these stakeholders, was that it had helped clients to get a better understanding of their barriers and possibilities for RTW.

*Labor expert: “I found it very surprising how clients already had made some important steps in the time between the meeting I had with them for the identification of RTW obstacles and subsequently the brainstorm session in which they discussed solutions to overcome these obstacles with the RTW coordinator, because it was clear for them what was the core of their problems and which of their problems they could influence.”*

The clients did not mention these functions when they reflected on the counselling they had received by the SSA. From their perception, obstacles for RTW and solutions to overcome these obstacles had barely been discussed. They thought that they had received not enough counselling by the SSA, as was explained by one of the clients:

*Client 2: “They assessed my capabilities and such. They also contacted the vocational rehabilitation agency. Then that part of the program started. And if there were any questions, I could contact them. But these did not really occur. We simply proceeded with the program and I did not receive any further support.”*

#### Perceived functions of direct placement in a competitive job

All clients indicated that their participation in the participatory supportive RTW program did not result in RTW in a competitive job. Also the other stakeholders confirmed that the program in many cases did not have the intended result, as many of their clients were not placed in competitive employment.

When the case managers of the vocational rehabilitation agencies were asked what actually had been done after referral of the client to their agency, they explained that they had put in a lot of effort to place the clients in a suitable job. They stressed that they did more than solely job hunting, e.g., helping the client with the writing of their CV and preparation for a job interview.

When describing the support they had received from the vocational rehabilitation agencies, some clients told that the case manager had not taken their job preferences into account and they had been treated like numbers. Others indicated that they had been in regular contact with the case manager who had helped them with their CV and application letters. Some mentioned that the case manager also had contacted companies to look for job opportunities. As a result, some clients were more positive than others. Still, most of them emphasized that they had received too little support from the case managers.

*Client 1: “I had one meeting with her and she would set to work. Finally she called me and said: ‘I never hear anything from you’. I asked her: ‘But shouldn’t I hear something from you?’ Actually, I did not understand anything of it.”*

The RTW coordinators of the SSA were responsible for monitoring the implementation of the RTW action plan. They also thought that the quality of the contracted vocational rehabilitation agencies differed a lot. They were dissatisfied with one agency, because of poor communication and involvement of this agency, but satisfied with another agency, because this agency started their job search very early after referral of a new client to their agency.

When clients were asked about their own participation in the program, they often indicated that they had been looking for job opportunities by themselves and had applied for several vacancies. They all wished to return to work. Some had found a voluntary job.

The case managers were quite positive about the cooperation by the clients in the search for a suitable job. However, in some cases they thought that the client could have participated more actively.

### Perceived barriers for a successful execution of the participatory supportive RTW program

#### Poor collaboration between the Dutch SSA, the vocational rehabilitation agencies and the (mental) healthcare sector

The professionals of the SSA mentioned several barriers that were related to a poor collaboration between their service and the contracted vocational rehabilitation agencies and/or the mental healthcare sector.

One of the insurance physicians explained that, because of segregation of services by the SSA and the healthcare sector, it was sometimes difficult to get in touch with the clients’ healthcare providers.

*Insurance physician: “That could be very time consuming. Some of the healthcare providers I could not reach by telephone. I even did not get their numbers. Once, the assistant of a general practitioner did not want to give me the number of a healthcare provider, because she was not sure that I was who I said that I was.”*

Another example of a poor collaboration mentioned by the SSA professionals was that the vocational rehabilitation agencies often analysed obstacles and solutions for RTW, while this was already done by the SSA. During the application of a participatory approach at the SSA, an action plan for RTW was made. From the perspective of the SSA professionals, the RTW action plans were useful in the search for a competitive job, as these summarized the most important obstacles for RTW, preconditions for RTW and suitable work. However, they thought that the agencies made only little use of the information in these action plans. This was confirmed by the case manager of one of the contracted vocational rehabilitation agencies, who explained that it could sometimes be necessary to use a broader perspective:

*Case manager of a vocational rehabilitation agency: “We talked with our client about what was discussed with the SSA, to see if this was still applicable. That was often the case. Sometimes we also considered other possibilities than the ones discussed by the SSA, so that we could use a broader perspective for our search. This was sometimes necessary, because we had to take the limited opportunities in the labor market into account.”*

The RTW coordinators of the SSA also admitted that, mainly due to time constraints, after referral of the client to the vocational rehabilitation agency they often had only limited contact with the client and barely monitored the actual implementation of the RTW action plans.

#### Type of (health) complaints

According to all stakeholders the type of (health) complaints experienced by the clients sometimes hampered a successful execution of the participatory supportive RTW program. In other words, the participatory supportive RTW program was not always considered suitable. In case the program was not seen as appropriate, this was often related to the perceived severity of the client’s (mental health) problems.

The insurance physicians indicated that for clients with severe (mental) health problems, participation in the program early after sick-listing could be too demanding, because of its intensity and early focus on work, and they were afraid that it would worsen their complaints.

*Insurance physician: “Often it concerned more complex cases, clients who needed attention on multiple aspects to improve functioning. In those cases, the program would have counteracted its purpose, because having to visit different professionals who ask different things would have been too demanding and intensive. It seems easy, but for some this is a huge task.”*

One of the RTW coordinators emphasized that for clients who participated in the participatory supportive RTW program it could be very difficult to point out obstacles for RTW, as a consequence of their mental health problems:

*RTW coordinator: “I believe that when you have serious mental health complaints, you can’t think clearly anymore. You don’t know exactly what has caused your complaints and what your capabilities are.”*

Mentioned by both labor experts and RTW coordinators was the difficulty to come up with solutions for obstacles for RTW, when these obstacles were related to the experienced mental health problems.

*Labor expert: “When someone has psychological problems it is more difficult to find a solution, then when someone faces a more concrete return to work obstacle”*

Also the placement in a competitive job was according to many stakeholders sometimes hampered by characteristics of the clients, such as an older age, a large distance to the labor market, a lack of application skills, passivity and mental health problems. According to some, this could lead to feelings of uncertainty, which formed another major obstacle for RTW.

*Client 4: “You have to compete with the rest of the world, while your own perception is that you’re not capable enough. That’s like being placed inside a boxing ring, together with professional boxers, while you’re still nothing.”*

*Case manager of a vocational rehabilitation agency: “…not searching for vacancies, because they were so insecure about their own capabilities that it complicated their job search. Every time they asked themselves: ‘Am I capable enough?’.”*

One of the case managers believed that the presence of mental health problems sometimes resulted in passivity and a lack of motivation. According to this case manager some clients also placed great demands on a vacancy, which made it difficult to find a suitable job.

Some of the professionals doubted if the client was ready for RTW, given his or her mental health problems. They thought that these clients needed more training prior to placement in a competitive job, such as training in empowerment or application skills, to increase their confidence, skills and motivation.

*RTW coordinator: “To be able to return to work, sometimes an increase of their mental resilience was necessary.”*

#### Time constraints

All stakeholders indicated that a lack of time was an important barrier for a successful execution of the program.

The labor experts and RTW coordinators stressed that the application of a participatory approach was very intensive. On the one hand this gave them the opportunity to get a full understanding of the client at an early stage and to gain the client’s confidence in their counselling. On the other hand, it was time consuming and asked a lot from both professionals and clients.

An important obstacle mentioned by all stakeholders was a lack of capacity at the SSA or vocational rehabilitation agency, often resulting in limited time to execute the program.

*RTW coordinator: “The workload at our department was high and we had to achieve several targets. Because there were no performance indicators for our participation in the Co-WORK study, often this work was done after other work was finished.”*

As was illustrated in this last quote, the limited capacity was partly related to the study setting. The participatory supportive RTW program was not part of the daily practice of the professionals and there were often other competing priorities. Moreover, only a few professionals in the organizations were trained in the program. In this way, it was not always possible to schedule all steps of the program in accordance with the prescribed time frame, as was explained by one of the labor experts:

*Labor expert: “In this way, every team member has to be available all the time. The workload was very high.”*

The case managers found that the period of 2 to 3 months in which they had to place the client in a suitable competitive job was too short, especially when the client was still facing mental health problems.

*Case manager of a vocational rehabilitation agency: “Given the problems of some people, or actually the majority of the people, a time-frame of 2 to 3 months appeared to be rather short sometimes. You want to get someone back on track very quickly, while sometimes there are serious complaints that get in the way and that need more attention.”*

Also many clients stressed that the received support for their job search was too short in time.

#### Limited labor market opportunities

A frequently mentioned barrier was the limited availability of suitable paid jobs in the Dutch labor market during the execution of the program, caused by the economic recession at the time.

The labor experts and RTW coordinators thought that in this situation it was difficult to think of suitable work. A RTW coordinator explained that it was often difficult to convert the preconditions for RTW into a concrete job:

*RTW coordinator: “You can wish to work on your own, because you can’t work together, or to get only one task at a time, or to have a break every ten minutes. Then you have figured out how you could function, but when you present these wishes to an employer, it is not realistic to think that they will offer you a job. Sometimes these work solutions may have been helpful, but they were not realistic to present to an employer.”*

Many stakeholders acknowledged that the clients often had to compete with a large number of other job seekers and many of them believed that an employer was not willing to hire an employee who is not fully employable.

### Perceived facilitators for a successful execution of the participatory supportive RTW program

#### Diminishing capacity needed

A facilitating factor mentioned by the labor experts was diminishing the number of professionals involved in the program, for example by letting the RTW coordinator perform all steps of the participatory approach. According to them, in this ways the capacity problem could be tackled.

One of the RTW coordinators and one of the case managers thought that involvement of the vocational rehabilitation agency in the development of the RTW action plan could have facilitated the search for a suitable job. Because of their knowledge of the labor market, the case managers could have helped matching the clients’ wishes and preconditions for RTW with opportunities in the labor market.

#### Creating opportunities in the labor market

Some clients, and also a few professionals, indicated that it would also have helped if the vocational rehabilitation agencies had already made some work arrangements with employers, including arrangements regarding therapeutic or sheltered workplaces. They stressed the importance of work and also of voluntary work, which could serve as a stepping-stone to more sustainable employment and help clients to become more self-confident.

## Discussion

### Main findings

The aim of this study was to gain insight into the execution of the participatory supportive RTW program within the Dutch social security sector, by evaluating stakeholders’ perceptions of the function(s) of the program, and their perceptions of barriers and facilitators for a successful execution. The findings of our study reveal that according to the professionals of the Dutch SSA, the functions of two components of this program – integrated care and a participatory approach – were as intended. These functions were respectively improving the communication and cooperation with the clients’ healthcare provider(s) to avoid conflicting advice about the clients’ possibilities for RTW, and making a consensus-based RTW action plan. However, the clients did not mention these functions. Instead, most of them stressed that they had received too little support from the SSA. Furthermore, both professionals and clients indicated that the job search based on the RTW action plan often did not result in placement of the client in a suitable competitive job. The execution of the program in the study’s practice appeared to be often not proceeded as intended. Several barriers for a successful execution of the full program were mentioned by the stakeholders. These barriers were related to a poor collaboration between the SSA, the vocational rehabilitation agencies and the mental healthcare sector, the type of (health) problems experienced by the clients, time constraints for the professionals, and limited opportunities at the Dutch labor market. Perceived facilitators for a successful execution of the program were: diminishing the number of SSA professionals involved, earlier involvement of the vocational rehabilitation agency, and making work arrangements with employers.

### Interpretation of findings

The use of a participatory approach had been positively evaluated in the previous process evaluation [5]. This could be explained by the perceived function of this component according to the professionals who applied this approach, which was in accordance with the intervention protocol. The perceived barriers for a successful execution of the full participatory supportive RTW program may help to explain the low number of sick-listed workers that was considered suitable for participation of the program and the overall low adherence to the protocol [5].

An important barrier, mentioned by many stakeholders, was the limited availability of suitable jobs in the labor market. This barrier was seen before in studies evaluating the feasibility and effectiveness of supported employment in the Netherlands and other European countries [18, 19]. Related to this barrier was the reluctance of Dutch employers to hire an employee with (mental) health problems, as perceived by some of the stakeholders. The same barrier was identified in the study of Van Erp et al. [19], who explained that because of a high level of employment protection in the Netherlands, hiring a worker with health problems implies a risk for the employer. Respondents in our study assumed that employers would not take this risk when there were also other candidates without health complaints.

The perceived barriers related to the type of (health) complaints, illustrate that there is still a very cautious approach regarding an early RTW of persons with a CMD. Although ‘place-and-train’ interventions such as supported employment have received growing attention in the last few years [18–20], in Europe the most common approach is still to ‘train-and-place’ in (sheltered or volunteer) work, with the emphasis on prevocational training [18, 19]. This may explain why stakeholders of the participatory supportive RTW program stuck to this approach.

The difficulties in the collaboration between the SSA, vocational rehabilitation agencies and the mental healthcare sector mentioned by professionals in our study illustrate how, despite attempts made for a better integration, this remained limited. This limited integration of services can be explained by comparing the participatory supportive RTW program with other RTW interventions. When we look at RTW interventions in which an integrated care approach was applied successfully [18, 20, 21], we see an early involvement of vocational services, and an integration of healthcare services and vocational services in one team of professionals. In the participatory supportive RTW program, the making of a RTW action plan, coordinated by the SSA, and placement in a suitable job, executed by vocational rehabilitation agencies, were organized as consecutive instead of integrated steps. Moreover the organization of (mental) health services and vocational services remained parallel.

The different perceptions of functions of the participatory supportive RTW program by clients and professionals of the SSA was in line with previous research. The study of Maiwald et al. [14] revealed that clients and professionals perceived the effectiveness of a RTW intervention differently because they focused on different outcomes. The clients who were interviewed in our study stressed that they wished to RTW. However, their participation in the program had not resulted in RTW in a competitive job. This might explain why according to them, the program did not have the intended function. The professionals of the SSA seemed to focus also on other outcomes, such as and active participation of the sick-listed worker. This might explain their more positive evaluation.

### Strengths and limitations of this study

An important strength of this study is that members of all stakeholder groups were interviewed. This made it possible to look for differences and similarities between perceptions of these different stakeholders of the execution of the participatory supportive RTW program in practice. This helped to get a full understanding of functions of the program according to these different stakeholders, and their perceptions of barriers and facilitators for a successful execution.

Another strength of this study is that the coding system was developed by two researchers, which increases the credibility of the analysis. However, the co-authors of this paper were only involved in the last phase of coding, i.e., selective coding. This can be seen as an important limitation of this study.

Another limitation of this study was that all interviews were held by telephone. This method was chosen, because we expected that both clients and professionals were more eager to participate and to talk freely when they could participate via a telephone conversation. However, non-verbal communication was not visible for both the interviewer and the interviewee, which is an important limitation of this method.

The non-response among some clients could be seen as another limitation. This could have biased our findings, as clients that agreed to participate might differ in perceptions from the ones who did not agree. However, the application of purposive sampling helped us to include clients with various characteristics.

Non-response among professionals was low. Nevertheless, input of one contracted vocational rehabilitation agency was missing, as its case manager was no longer working for this agency and we were not able to contact him. Furthermore, professionals of only four SSA offices participated in the present study, whereas in total seven SSA offices had participated in the Co-WORK study. This was caused by the very low number of cases in which the participatory supportive RTW program had been applied at the remaining offices. By selecting professionals that applied the program at least twice, we may have selected professionals who were more willing to implement the program. Still, also the selected professionals had applied the program only a few times. The number of clients that had actually participated in the intervention was very low (*N* = 36) [5]. This means that the number of cases per professional in which the program was applied was also low. Both the applied selection of professionals, and the low number of cases per professional could have biased our findings and can be considered as important limitations of this study.

The timing of the interviews can be seen as another cause of possible bias and forms another limitation of this study. All clients had started with the program more than 1 year before the interview. This might have resulted in recall bias.

### Implications for practice and research

Our findings emphasize the need for a better integration of services from the Dutch SSA, vocational rehabilitation agencies and from the mental healthcare sector, in order to respond to the (vocational) needs of workers without a (permanent) employment contract, sick-listed due to a CMD. An important point of improvement mentioned by the respondents in this study, is an earlier involvement of vocational rehabilitation agencies in RTW counselling.

Furthermore, lessons may be learned from supported employment [22]. In this evidence-based approach employment specialists and healthcare providers cooperate in order to search for a suitable job as quickly as possible, and to support their client during work resumption for as long as needed. Until now, the focus of studies evaluating this practice has been almost exclusively on people with severe mental illnesses [22]. It seems worthwhile to investigate whether a similar collaboration is effective in improving RTW for people with less severe and more common mental health problems, by simultaneously addressing treatment and vocational needs.

To stimulate a successful integration of mental healthcare in vocational rehabilitation of workers without a (permanent) employment contract who are sick-listed due to a CMD, it seems important that employment problems and outcomes become central in the treatment of mental health problems [22]. In this regard, the recently signed covenant between the Dutch SSA and mental healthcare sector could be seen as an important step forward. Possibly, this covenant could be taken as a starting point to stimulate further integration of services.

We recommend to evaluate in future research whether more intensive and earlier involvement of vocational rehabilitation agencies and mental healthcare providers would help to identify barriers for RTW in an early phase, and to better match the sick-listed worker with (the limited) opportunities in the labor market.

## Conclusions

The results of this study indicate that, despite the quite positive evaluation of the functions of integrated care and a participatory approach, there were multiple barriers for a successful execution of the full participatory supportive RTW program. Execution of the program seemed to be highly influenced by the limited availability of suitable jobs in the Dutch labor market, the belief of some professionals that an early RTW of sick-listed workers with mental health problems should be avoided, the segregation of services within the Dutch social security sector, and by time constraints for professionals. For future implementation of the program in the Dutch social security sector, it will be important to consider how integration of services by the Dutch SSA, vocational rehabilitation agencies and the mental healthcare sector can be improved in order to respond to the (vocational) needs of sick-listed workers with a CMD.

## Availability of data and materials

Data and materials for this study are available through communication with the corresponding author of this manuscript, F.G. Schaafsma.

## Additional file

Additional file 1: ‘Execution of a Participatory Supportive Return to Work Program within the Dutch Social Security Sector: a Qualitative Evaluation of Stakeholders’ Perceptions in BMC Public Health. (DOCX 19 kb)

## Abbreviations

CMD: Common mental disorder
OHC: Occupational health care
RCT: Randomised controlled trial
RTW: Return to work
SSA: Social Security Agency
