# Supplementary material for: Execution of a participatory supportive return to work program within the Dutch social security sector: a qualitative evaluation of stakeholders’ perceptions
Source: BMC Public Health. 2016 Apr 14;16:323. doi: 10.1186/s12889-016-2997-x (PMC4831193; doi:10.1186/s12889-016-2997-x)
Supplement: Additional file 1: — ‘Execution of a Participatory Supportive Return to Work Program within the Dutch Social Security Sector: a Qualitative Evaluation of Stakeholders’ Perceptions in BMC Public Health. (DOCX 19 kb) [file 12889_2016_2997_MOESM1_ESM.docx]

**Additional file 1.**

*Additional material to article by L. Lammerts, F.G. Schaafsma*^1^*, W. van Mechelen and J.R. Anema ‘Execution of a Participatory Supportive Return to Work Program within the Dutch Social Security Sector: a Qualitative Evaluation of Stakeholders’ Perceptions’ in BMC Public Health*

^1^Department of Public and Occupational Health, EMGO+ Institute for Health and Care Research, VU University Medical Center. E-mail: [f.schaafsma@vumc.nl](mailto:f.schaafsma@vumc.nl)

**Table A1. Topic-list**

| Topics | Discussed with: |
| --- | --- |
| *General:* |  |
| Experience with participation in the intervention | All respondents |
| Satisfaction with intervention | All respondents |
| Ideas about an early RTW of sick-listed workers with a CMD | All respondents |
| Effectiveness of intervention | All respondents |
| Points of improvement of intervention | All respondents |
| Relationship between intervention and expectancies beforehand | Clients |
| Relationship between intervention and needs for RTW guidance | Clients |
| Own role in conduct of intervention | Professionals |
| *Integrated care:* |  |
| Experience with guidance by insurance physician | Clients |
| Ability to work according to insurance physician and own ideas about ability to work | Clients |
| Advice on RTW by insurance physicians and healthcare providers | Clients |
| Experience with contacting healthcare providers | Insurance physicians |
| Effects of contacting healthcare providers | Insurance physicians |
| *Participatory approach:* |  |
| Development of RTW action plan | Clients, labor experts,  RTW coordinators |
| Identification of obstacles for RTW | Clients, labor experts,  RTW coordinators |
| Thinking about solutions to overcome RTW obstacles | Clients, labor experts,  RTW coordinators |
| Thinking about suitable work | Clients, labor experts,  RTW coordinators |
| Relationship between health complaints and thinking about obstacles for RTW, solutions to overcome obstacles, and suitable work | Clients, labor experts,  RTW coordinators |
| Own role in development RTW action plan | Clients, labor experts,  RTW coordinators |
| Implementation of RTW action plan | Clients, labor experts,  RTW coordinators |
| Looking for a job, based on RTW action plan | Clients |
| Experience with guidance by RTW coordinator | Clients |
| Experience with guidance by labor expert | Clients |
| Participation of client in developing RTW action plan | Labor experts, RTW coordinators |
| Collaboration between stakeholders in developing RTW action plan | Labor experts, RTW coordinators |
| *Direct placement in a competitive job:* |  |
| Support by vocational rehabilitation agency in job search | Clients |
| Effectiveness of job search by vocational rehabilitation agency | Clients, case managers |
| Effectiveness of own job search | Clients |
| Collaboration between professionals of SSA and of vocational rehabilitation agencies | Clients, RTW coordinators, case managers |
| Ways in which was searched for a suitable job | Clients, case managers |
| Ways in which a suitable job was offered to client | Clients, case managers |
| Collaboration with client in job search | Case managers |
| *Other:* |  |
| Contra-indications for participation in intervention | Insurance physicians |
| Delay in execution of intervention | Labor experts, RTW coordinators |
